# Low-dose alemtuzumab induction in a tailored immunosuppression protocol for sensitized kidney transplant recipients

**DOI:** 10.1186/s12882-020-01767-z

**Published:** 2020-05-13

**Authors:** Martina Guthoff, Kilian Berger, Karina Althaus, Thomas Mühlbacher, Tamam Bakchoul, Wolfgang Steurer, Silvio Nadalin, Alfred Königsrainer, Nils Heyne

**Affiliations:** 1 Department of Diabetology, Endocrinology, Nephrology, Section of Nephrology and Hypertension, University of Tübingen, Otfried-Müller-Str. 10, 72076 Tübingen, Germany; 2 Institute for Diabetes Research and Metabolic Diseases of the Helmholtz Center Munich at the University of Tübingen, Otfried-Müller-Str. 47, 72076 Tübingen, Germany; 3 German Center for Diabetes Research (DZD e.V.), Neuherberg, Germany; 4 Center for Clinical Transfusion Medicine, Otfried-Müller-Str. 4/1, 72076 Tübingen, Germany; 5 Department of General- and Visceral Surgery, Leonberg Hospital, Rutesheimer Str. 50, 71229 Leonberg, Germany; 6 Department of General-, Visceral- and Transplant Surgery, University of Tübingen, Hoppe-Seyler-Str. 3, 72076 Tübingen, Germany

**Keywords:** Kidney transplantation, HLA-sensitization, Induction, Protocol, Alemtuzumab, Maintenance immunosuppression, Allograft survival, Rejection, Infection

## Abstract

**Background:**

Induction therapy is crucial in kidney transplantation and constitutes an important cornerstone for long-term allograft survival. Alemtuzumab is a depleting CD52-specific antibody with T- and B-cell activity, leading to prolonged lymphocyte depletion for up to 12 months, with profound immunosuppression and an associated risk of serious infections. Current concepts aim to optimize dosing strategies to reduce side effects. Here we present data from an ongoing centre protocol consisting of low-dose alemtuzumab induction and tailored immunosuppression in sensitized patients undergoing kidney transplantation.

**Methods:**

10-year results of the protocol were analysed. Low-dose alemtuzumab induction consisted of a single dose of 20 mg intraoperatively, followed by tacrolimus and corticosteroids for initial immunosuppression, with mycophenolate mofetil suspended until a total lymphocyte count (TLC) >5% or 200/μl was reached.

**Results:**

Between 01/2007 and 04/2017, 46 patients were treated in accordance with the protocol in 48 kidney transplantations. Median PRA_max_ was 43 [22-76; IQR] %; all patients had negative CDC-crossmatch prior to transplantation. Low-dose alemtuzumab was well tolerated. Median time to TLC recovery was 77 [62-127; IQR] d. Within a median follow-up of 3.3 [1.5-5.6; IQR] years, 12 (25%) patients developed BPAR, 10 of which were antibody-mediated (3 acute, 7 chronic ABMR). Death-censored 5-year allograft survival was 79.2%, with an excellent allograft function at the end of follow-up. There was no increased rate of infections, in particular viral infections.

**Conclusions:**

Our protocol, comprising low-dose alemtuzumab induction, initial suspension of mycophenolate mofetil and triple maintenance immunosuppression, provides excellent patient and allograft outcome in sensitized renal allograft recipients.

## Background

Induction therapy is crucial in kidney transplantation and constitutes an important cornerstone for long-term allograft survival. Induction is used to cover the immediate post-transplant phase as the period with the highest risk of acute rejection. Nevertheless, choice of induction regimen also has an impact on the risk for later development of donor-specific antibodies (DSA) and late allograft rejection [1–3].

Sensitized patients with pre-existing HLA-antibodies are at high risk of acute and chronic antibody-mediated rejection [4] and constitute a major challenge in kidney transplantation. HLA-sensitization occurs via contact with allo-antigens due to pregnancy, blood transfusion or previous transplantations, the latter being the most immunogenic with the lowest allograft survival in subsequent transplantation [5].

Two different classes of agents are used for induction therapy: non-depleting antibodies, such as CD25 inhibitory antibodies (directed against the α-chain of interleukin 2 (IL2) receptor), which block IL2-mediated T-cell stimulation, and depleting antibodies, which lead to total lymphocyte depletion and include antithymocyte globulin (ATG) and the CD52 antibody alemtuzumab. Depleting antibodies have higher immunosuppressive potential than CD25 inhibitory antibodies [6–8]; however, associated concerns include over-immunosuppression with the risk of infection and other related side effects.

Alemtuzumab, a humanized monoclonal antibody directed against CD52 on B- and T-lymphocytes, monocytes and NK cells, is used in the treatment of lymphoma and multiple sclerosis [9]. Alemtuzumab has been used for induction in kidney transplantation since 1998 [9]. Compared to ATG, alemtuzumab, when administered in standard doses of 30–60 mg, results in the same or even better efficacy with regard to rejection episodes [3, 6, 10–12]. Complete B- and T-lymphocyte depletion, however, persists much longer than with ATG [13], which is accompanied by an increased risk of infection [14]. The dosage for induction in kidney transplantation was historically chosen arbitrarily and pharmacokinetic studies in this indication are lacking [9]. The rationale for a reduced dose of alemtuzumab for induction was to exploit its beneficial effect whilst reducing the period of lymphopenia with associated side effects. In 2007, we implemented an induction protocol using low-dose alemtuzumab and specifically tailored immunosuppression in sensitized kidney allograft recipients. The aim was to establish a centre protocol to balance immunosuppression and its associated side effects in this high risk patient population.

## Methods

### Aim, design and setting of the study

All renal transplant recipients treated according to the centre induction protocol in kidney or simultaneous pancreas-kidney transplantation between 01/2007 and 04/2017 at the Tübingen University Hospital Collaborative Transplant Centre were included in the analysis. Children (below 18 years of age) were excluded. Data was analysed retrospectively. The retrospective analysis was conducted in accordance with the Declaration of Helsinki and approved by the local institutional review board (482/2016BO2).

### Patients and induction protocol

HLA-sensitized patients were treated according to the centre protocol if their maximum panel reactive antibodies (PRA) were ≥ 15% in deceased donor transplantation or if they had received HLA-incompatible living donor transplantation, defined as the presence of DSA prior to transplantation. Preconditioning in the latter comprised desensitization with rituximab and immunoadsorption or plasmapheresis. A single plasmapheresis was also performed in the majority of patients prior to deceased donor transplantation. A negative complement-dependent cytotoxicity crossmatch was required in all patients prior to proceeding with transplantation.

**Fig. 1:**
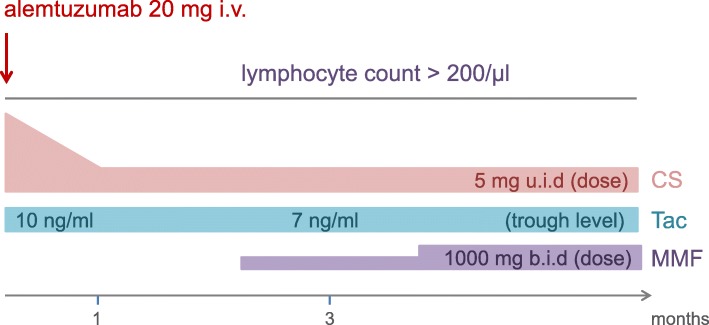
Induction protocol with low-dose alemtuzumab and tailored immunosuppression. Tac: tacrolimus, MMF: mycophenolate mofetil, CS: corticosteroids

The protocol consisted of low-dose alemtuzumab for induction (20 mg intravenously prior to reperfusion) and tailored immunosuppression, commencing with tacrolimus (aiming at trough levels of 10 ng/ml initially and 7 ng/ml from month three on) and corticosteroids (steroid pulse starting with 500 mg and 250 mg prednisolone intravenously at time-point of transplantation and 12 h later, respectively, followed by 100 mg u.i.d given orally with taper to 5 mg u.i.d. within 1 month). Mycophenolate mofetil (MMF) was suspended until lymphocyte count in peripheral blood reached > 5% of leukocytes or > 200/μl absolute (dose increments aiming at 1000 mg b.i.d., depending on clinical tolerance), resulting in triple maintenance immunosuppression (Fig. 1). *Pneumocystis jirovecii* prophylaxis was administered for 6 months, prophylaxis for cytomegalovirus infection for 3–6 months (depending on mismatch constellation) after transplantation.

### Parameters

The following parameters were assessed: patient characteristics, number of previous transplants, recipient immunology, type of transplantation (deceased vs. living donor, combined transplantation), donor characteristics, data on transplant and induction procedure, immediate graft function, time to lymphocyte recovery, duration of follow-up, graft function, development of DSA, biopsy-proven acute rejection (BPAR), infectious complications and posttransplantation diabetes mellitus.

### Immunology

Patients’ sera were investigated for HLA class I and II antibodies using the Luminex technology starting in 2009 (LABScreen and single antigen flow bead assays, One Lambda, Canoga Park, CA, USA). Data on immunology included PRA at the time of transplantation and maximum PRA (calculated by using the unacceptable antigens, with all complement-fixing antibodies for class I and all donor-specific antibodies for class II before 2009, and single antigen assay after 2009, with all antibodies added to the PRA when belonging to a DSA-related specific cross-reactive group in class I and all specific antibodies for class II above a mean fluorescence intensity of 5000), number of unacceptable antigens, number of mismatches in HLA class I and II, and the development of DSA during follow-up. HLA antibody determinations post transplantation was performed by clinical indication. BPAR was defined as T-cell mediated rejection (TCMR) or antibody-mediated rejection (ABMR) in accordance with BANFF criteria in kidney biopsy samples [15]. Borderline changes only were not counted as BPAR.

### Infectious complications

Infectious complications were classed into viral (cytomegalovirus, Epstein-Barr virus, polyomavirus), bacterial (with focus on urinary tract infection) and other infections. Screening for BK viraemia was performed in an incidence-based manner upon worsening of allograft function.

### Statistical analysis

Unless otherwise indicated, data are given as median [interquartile range]. Kaplan-Meier curves were generated for rejection-free allograft survival and allograft survival by calculating the probability for the event for each time point, taking censored patients into consideration. All statistics were performed using the JMP 13.1.0 (SAS Institute, Cary, NC) statistical software package.

## Results

Between 01/2007 and 04/2017, 46 patients were treated according to the induction protocol at the Tübingen Collaborative Transplant Centre. Two of these patients were transplanted twice during the observation period, resulting in a total of 48 transplantations. Further analyses are based on n = 48 as a reference figure. Two kidneys were transplanted simultaneously with a pancreas. Median follow-up of patients was 3.3 [1.5-5.6] years with a maximum follow-up of 9.7 years.

### Patient and transplant characteristics

Patient and transplant characteristics are displayed in Table 1. Median age of patients at time of transplantation was 49 [45–57] yrs. In all, 16 patients presented for first transplantation, 20 for second and 12 for third or higher transplantation. Median PRA_max_ was 43 [22–76] %. Nine patients received HLA-incompatible living donor transplantation and were desensitized with rituximab (375 mg/m^2^ 4 weeks prior to transplantation) and between 0 and 14 sessions of immunoadsorption. Thirty-nine patients received deceased donor transplants, 34 of whom were treated with a single plasmapheresis prior to transplantation.

**Table 1:** Patient and transplant characteristics

patient characteristics	
gender (f/m)	27 / 21
age (yrs)	49 [45–57]
body weight (kg)	68 [59–78]
BMI^a^ (kg/m^2^)	22.9 [21.2–26.1]
# of transplantation (*n*)	
1st	16
2nd	20
3rd or more	12
PRA^b^ max. (%)	43 [22–76]
transplant characteristics	
DD / LD^c^	39 / 9
donor age (yrs.)	52 [44–58]
MM HLA class I (A + B)^d^ (*n*)	2 [2–3]
MM HLA class II (DR + DQ)^d^ (*n*)	2 [1–2.3]
CIT^e^ (h)	10.2 [5.0–15.1]
PP/IA^f^ prior to transplantation (y/n)	42 / 6

Data are given as median [interquartile range]

^a^body mass index; ^b^PRA: panel reactive antibody; ^c^DD: deceased donor transplantation, LD: living donor transplantation; ^d^MM: mismatches, HLA: human leukocyte antigen; ^e^CIT: cold ischemia time; ^f^PP: plasmapheresis, IA: immunoadsorption

Median body weight at transplantation was 68 [59–78] kg, resulting in an alemtuzumab dose of 0.30 [0.26–0.34] mg/kg. Alemtuzumab was generally well tolerated. Three patients (6%) suffered from transient allergic reaction/cytokine release after administration of alemtuzumab, which responded well to corticosteroid administration and vasopressors. Another three patients (6%) displayed haemorrhagic diathesis during transplantation and required additional haemostasis.

### Lymphocyte recovery

**Fig. 2:**
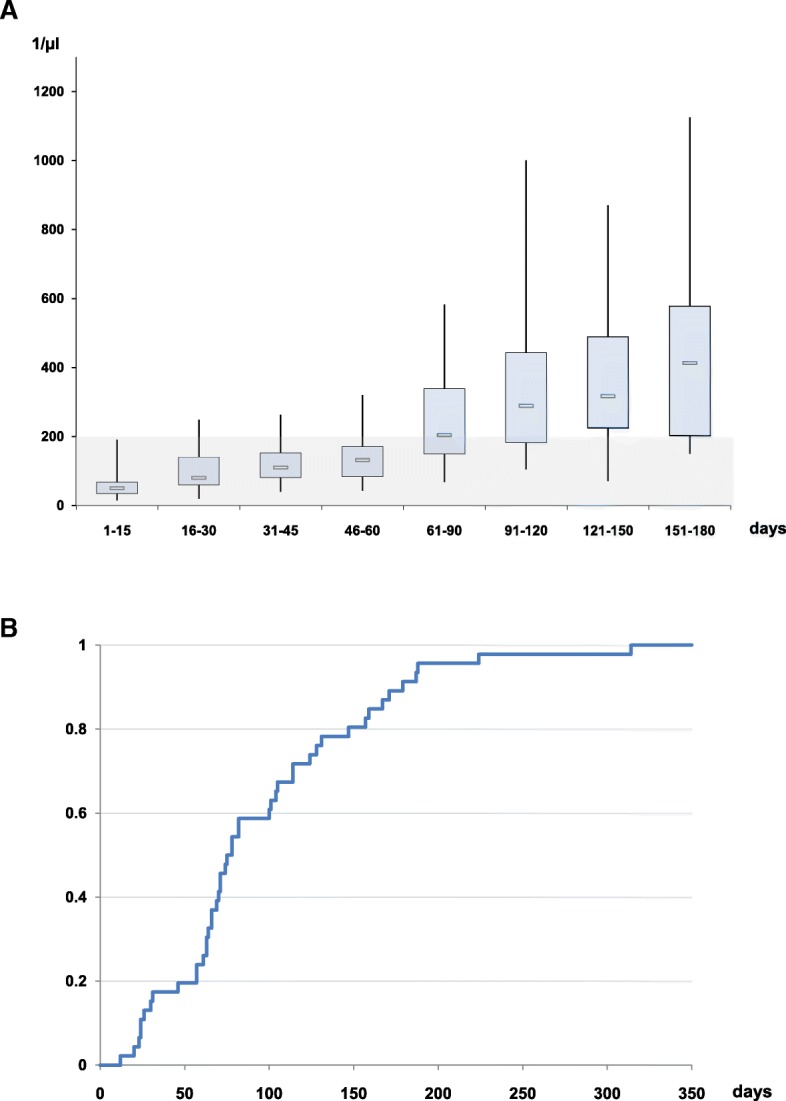
**a**: Lymphocyte count of patients at different time intervals. Box plots display median (interior bar), interquartile range (upper and lower margin of rectangle) and maximum/minimum (whiskers). **b**: Cumulative incidence of total lymphocyte recovery, defined as lymphocyte count in peripheral blood of > 200/μl

Time-course of lymphocyte recovery is depicted in Fig. 2a. Complete data on lymphocyte recovery was available from 46 patients. In peripheral blood, median time to lymphocyte recovery, defined as > 200 lymphocytes/μl, was 77 [62-127] days. Cumulative incidence of lymphocyte recovery is displayed in Fig. 2b.

With initiation of MMF, all patients stayed on triple maintenance immunosuppression during follow-up.

### Allograft function

Fourteen patients (29.2%), all recipients of deceased donor transplants, had delayed allograft function, defined as the need for dialysis within the first week after transplantation. None of the patients after living donor kidney transplantation experienced delayed graft function. Upon their discharge from hospital, all grafts were functioning with a median eGFR of 43 [30-61] ml/min/1.73 m^2^ (Table 2).

**Table 2:** Outcome

early posttransplant period	
delayed allograft function (*n*, %)	14 (29.2)
plasma creatinine at discharge (μmol/l)	141 [106–177]
eGFR^a^ at discharge (ml/min/1.73 m^2^)	43 [30–61]
lowest thrombocyte count (10^9^/L)	101 [79–132]
time to lowest thrombocyte count (d)	2 [1–3]
long-term follow-up	
follow-up (yrs.)	3.3 [1.5–5.6]
all-cause allograft loss (*n*, %)	14 (29.2)
time to allograft loss (yrs.)	2.1 [0.4–2.9]
death-censored allograft loss (*n*, %)	9 (18.8)
plasma creatinine* (μmol/l)	124 [106–150]
eGFR^a^ * (ml/min/1.73 m^2^)	47 [39–65]
BPAR^b^ (*n*, %)	12 (25)
TCMR (*n*, %)	2 (4)
ABMR (*n*, %)	10 (21)
urinary tract infections/patient/year (*n*)	0.7 [0.4–2.4]
viral infections (CMV, EBV)^c^ (*n*, %)	7 (15)
PVAN^d^ (*n*, %)	1 (2.1)
prediabetes / PTDM^e^ ** (*n*, %)	16 (38.1) / 13 (31.0)

Data are given as median [interquartile range]

^a^eGFR: estimated glomerular filtration rate (according to [16]); * of those with functioning graft; ^b^BPAR: biopsy proven acute rejection, TCMR: T-cell mediated rejection, ABMR: antibody-mediated rejection; ^c^CMV: cytomegalovirus, EBV: Epstein-Barr virus; ^d^PVAN: polyomavirus-associated nephropathy; ^e^PTDM: posttransplantation diabetes mellitus; ** of 42 patients without preexisting diabetes mellitus

**Fig. 3:**
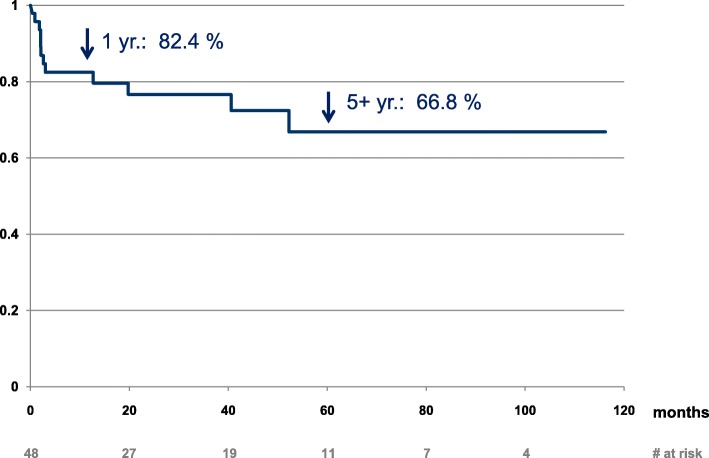
Kaplan-Meier estimate of rejection-free allograft survival

**Fig. 4:**
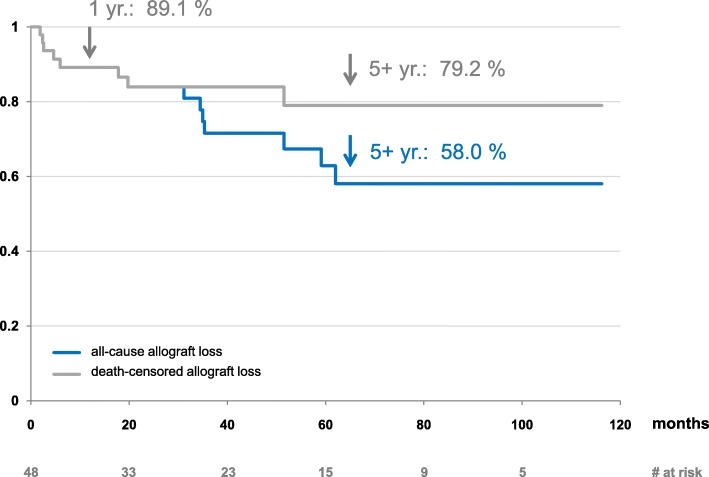
Kaplan-Meier estimate of allograft survival; blue line: all-cause allograft loss, grey line: death-censored allograft loss

During follow-up, 12 patients (25%) developed BPAR (Fig. 3), of which 10 cases were antibody-mediated. Only two of the rejection episodes were late rejections > 2 years after transplantation. During follow-up, 14 out of 48 allografts were lost, of which 6 were attributed to acute or chronic rejections, none of these occurred in HLA-incompatible living donor kidney transplantations. Five allografts were lost due to death with functioning allograft (Fig. 4). Causes of death were de novo malignancies (breast, pancreas and cancer of unknown primary), haemorrhagic shock due to severe gastrointestinal bleeding and heart failure. Deaths occurred between 31 and 62 months after transplantation. Other causes of allograft loss were poor organ quality, allograft pyelonephritis and one unknown cause. At the end of individual follow-up, eGFR in patients with allograft survival (*n* = 34) was excellent, with an eGFR of 47 [39-65] ml/min/1.73 m^2^ (Table 2).

### Development of de novo donor-specific antibodies

Screening for DSA post transplantation was performed when the clinical course of laboratory parameters was suggestive of allograft rejection. It was performed in 22 out of 48 transplantations, 10 of which were tested positive for de novo DSA against HLA class I (*n* = 5), HLA class II (*n* = 4) or both (*n* = 1).

### Infectious complications

Five patients (10.4%) developed cytomegalovirus infection during follow-up. One patient showed late polyoma BK-virus replication in blood and polyomavirus-associated nephropathy in biopsy 9 years after transplantation. Two patients were detected with EBV replication in blood. However, none of patients developed posttransplant lymphoproliferative disease (Table 2).

Sixteen patients did not suffer from any urinary tract infections whatsoever. In the remaining patients, 78 urinary tract infections were recorded during individual follow-up, resulting in a median infection rate of 0.7 [0.4-2.4] per patient/year.

A total of 40 other infectious episodes were recorded in the patients during long-term follow-up, including gastrointestinal or pulmonary infections. Pneumocystis jirovecii pneumonia and invasive fungal infection were not recorded in any patient.

### Glucose metabolism

Prior to transplantation, 6 out of 48 patients had pre-existing diabetes. Among the others, 38.1% developed prediabetes and 31% PTDM during follow-up, 27.1% of patients displayed normal glucose regulation (Table 2).

## Discussion

In immunosuppressive regimen, balancing the need for immunosuppression and the risk of associated infections is challenging, especially in cohorts with high immunological risk. In our study, we demonstrate an induction protocol with low-dose alemtuzumab and tailored immunosuppression to provide excellent outcome without increased rates of infection in sensitized kidney transplant recipients.

The success of our protocol is based on three factors: i) a reduced dose of alemtuzumab, ii) suspension of MMF until lymphocyte recovery and iii) triple maintenance immunosuppression.

The first aspect to be discussed is whether or not efficacy is compromised in comparison to standard alemtuzumab induction. Studies regarding the efficacy of alemtuzumab in induction therapy have yielded conflicting results and have not fostered confidence in the broader use of alemtuzumab in kidney transplantation in the past. While favourable rejection rates for alemtuzumab compared to non-depleting antibodies have been demonstrated [6–8], comparison with ATG is discussed more controversially. A randomized controlled trial with 222 patients demonstrated less BPAR with alemtuzumab [17], other studies demonstrated the contrary [18] or no significant difference [6]. Superiority has been shown in patients with increased immunological risk [3, 10]. The main difference in pharmacodynamics between ATG and alemtuzumab are target cells; ATG, on the one hand, is predominantly directed against T-cells and, by virtue of its polycloncal effect, also exerts moderate effects on B-cells [19]. Alemtuzumab, on the other hand, is directed against CD52, which is expressed on T-cells, B-cells, natural killer cells, dendritic cells and a number of others [9] and leads to a more profound impact on humoral immune response as well as on innate immunity. Therefore, it seems rational to use alemtuzumab, particularly in HLA-sensitized patients. However, one study, published in 2013, demonstrated increased B-cell reconstitution and subsequent DSA formation in patients treated with alemtuzumab [20], albeit the study included only a small number of such patients treated with alemtuzumab.

The dosage of alemtuzumab given for induction in kidney transplantation varies between 30 and 60 mg [21]. In our study, we use a low-dose approach of 20 mg administered once intraoperatively. With this scheme, all of our patients show immediate and complete lymphocyte depletion, as intended in this early post-transplant phase. Nevertheless, 12 of our patients experienced rejection and most were antibody-mediated. However, most were in the early post-transplant phase and responsive to treatment and long-term allograft survival was excellent, especially since there were only two late allograft rejections. Compared to published outcomes in HLA-sensitized patients after various desensitization protocols, our rejection rates were even lower (25% vs. 36%, as published in [22]). Bearing the immunological risk of the investigated cohort in mind, our results are encouraging. So, to answer our question, a reduced dose of alemtuzumab does not result in reduced efficacy.

With regard to infectious complications, the reduced dose yields better results than standard dosing. With doses of 30–60 mg of alemtuzumab, prolonged leukocytopenia and lymphopenia have been reported [3, 23]. The idea of dose adjustment was reported in an earlier study that used a dosage scheme depending on body weight (0.4 mg/kg). However, the median doses applied in this study were in the lower range of standard dosing and not significantly reduced [21]. Using a reduced dose of 20 mg, resulting in a median dose of 0.3 [0.26–0.34] mg/kg, we were able to demonstrate that time to lymphocyte recovery is markedly shorter than standard dosage [24]. With a median time to lymphocyte recovery of 77 days, the critical period of being prone to infections was kept short without putting the patients at immunological risk. As a consequence, the overall infection rate demonstrated in our study was very low, particularly with regard to viral and opportunistic infections. CMV infection was documented in 10% of patients, compared to 27% in a study by Margreiter and colleagues [25]. Increased rates of polyomavirus-infections have also been previously observed [8] but could not be confirmed in our study, although our low number of polyomavirus-infections could in part be attributed to the fact that, during the period of observation, we performed incidence-based screening only. We had no cases of *Pneumocystis jirovecii* or invasive fungal infection, nor did any of the patients develop PTLD. In this context, suspension of MMF until lymphocyte recovery, as in our protocol, might further contribute to low infection rate.

The third factor, triple maintenance immunosuppression, is key for long-term success. Many trials investigating alemtuzumab induction were designed to reduce maintenance immunosuppression. Higher late rejection rates with alemtuzumab observed in some studies may be attributed to the minimization of maintenance immunosuppression [18, 26]. With our concept of triple maintenance immunosuppression, we demonstrate a low rate of late rejections (two rejections > 2 years of follow-up) and excellent long-term graft survival with almost 80% of functioning grafts after 5 years at an excellent eGFR, an allograft survival rate markedly better than published in patients with HLA-sensitization prior to transplantation [27].

Finally, we demonstrated a high overall rate of prediabetes and post-transplantation diabetes mellitus in our patients, a finding which had not been detected in previous studies [6, 25]. Since alemtuzumab has not been described as having diabetogenic potential in the literature so far, and good results have been achieved when it is used for induction in pancreas or islet transplantation [28, 29], we attribute this effect to dosage and duration of overall immunosuppression (e.g. corticosteroid maintenance therapy, higher target tacrolimus trough levels) in our risk cohort with a high rate of second or higher transplantation, rather than to an effect of alemtuzumab.

Our study does have limitations: As a retrospective analysis, we lack a comparator group for different induction regimes in the same patient population. Furthermore, the group is heterogeneous and also comprises patients with additional preconditioning prior to living donor kidney transplantation. We do, however, show the long-term experience with a standardized and balanced induction protocol in a large number of sensitized patients of a university hospital, a cohort representative for many European transplant centres.

## Conclusion

In conclusion, our protocol carefully balances the need for immunosuppression and the risk of infection, based on different phases after transplantation. The potent anti-CD52 antibody alemtuzumab covers the immediate post-transplant period, whereas the low-dose concept in combination with suspension of MMF reduces the associated short-term risk. Triple immunosuppression after lymphocyte recovery fosters long-term efficacy. Our results thereby pave the way for a second look at alemtuzumab induction, implemented in specific protocols, in kidney transplantation.

## Data Availability

With acceptance, a data file is archived in the Dryad Digital Repository. After discussion with the ethics committee, legal department and medical faculty, the data file has been restricted regarding patient demographic and anthropometric variables to provide anonymity. All measured values relevant to the analysis are included. Background to this is the fact that our cohort is strictly selected cohort from a single university hospital, hence an otherwise very closely described patient group.

## Abbreviations

ABMR: Antibody-mediated rejection
BPAR: Biopsy-proven acute rejection
CDC: Complement-dependent cytotoxicity
DSA: Donor-specific antibodies
eGFR: estimated glomerular filtration rate
HLA: Human leukocyte antigen
IL2: Interleukin 2
MMF: Mycophenolate mofetil
PRA: Panel reactive antibody
ATG: Antithymocyte globulin
TCMR: T-cell mediated rejection
TLC: Total lymphocyte count
